# Electrochemical Impedance Spectroscopy for Ion Sensors with Interdigitated Electrodes: Capacitance Calculations, Equivalent Circuit Models and Design Optimizations

**DOI:** 10.3390/bios14050241

**Published:** 2024-05-10

**Authors:** Eva-Maria Korek, Reva Teotia, David Herbig, Ralf Brederlow

**Affiliations:** School of Computation, Information and Technology, Technical University of Munich, 80333 Munich, Germanyda.herbig@tum.de (D.H.); r.brederlow@tum.de (R.B.)

**Keywords:** electrochemical impedance spectroscopy, solid-state ion sensor, interdigitated electrodes, equivalent circuit model, FEM simulations, ion-sensitive membrane

## Abstract

Electrochemical impedance spectroscopy (EIS) is becoming more and more relevant for the characterization of biosensors employing interdigitated electrodes. We compare four different sensor topologies for an exemplary use case of ion sensing to extract recommendations for the design optimizations of impedimetric biosensors. Therefore, we first extract how sensor design parameters affect the sensor capacitance using analytical calculations and finite element (FEM) simulations. Moreover, we develop equivalent circuit models for our sensor topologies and validate them using FEM simulations. As a result, the impedimetric sensor response is better understood, and sensitive and selective frequency ranges can be determined for a given sensor topology. From this, we extract design optimizations for different sensing principles.

## 1. Introduction

Ions are prevalent biomarkers and their quantification is important for a diverse field of applications ranging from healthcare and agriculture to food and water quality monitoring, as well as the control of industrial production applications [1]. In healthcare, e.g., potassium (K^+^) and sodium (Na^+^) ions serve as indicators for athletic performance such as hydration and muscular fatigue and diseases such as hyponatremia and hypokalemia [2]. Many of these application fields still lack portable low-energy monitoring systems. For this reason, we are developing cost-effective miniaturized sensors for the detection of ion concentrations in, e.g., water or sweat via electrochemical impedance spectroscopy (EIS).

EIS is a method used not only for sensor characterization and optimization [3,4,5,6] but also for the detection of analytes in gaseous and fluid environments [7,8,9,10,11,12,13]. In potentiostatic EIS, a sinusoidal voltage signal $E=E_{0}\sin\left(\omega t\right)$ is applied to the electrodes using single, multiple, or sweeping frequencies, and the corresponding current $I=I_{0}\sin\left(\omega t+\varphi \right)$ is measured using an LCR meter or a potentiostat [7,14]. The sensor impedance can be calculated by measuring the amplitude |Z| and phase shift φ of the sensor signal via [14]

(1) $$Z=\frac{E}{I}=\left|Z\right|e^{j\varphi }=\left|Z\right|\left(\cos\varphi +\mathrm{jsin}\varphi \right)=Z_{real}+jZ_{im}.$$

**Figure 1:**
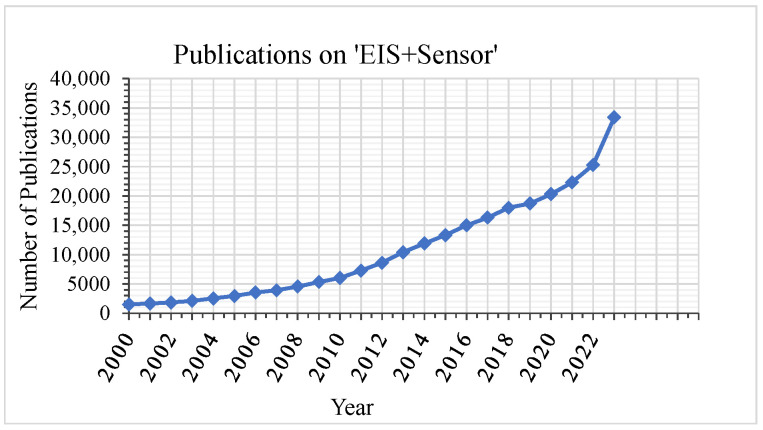
Number of publications containing “electrochemical impedance spectroscopy” and “sensor” in Google Scholar.

For impedimetric sensors, interdigitated electrodes (IDEs) are commonly used as a transducer element due to their advantages of low cost and simple fabrication. As a consequence, these electrodes can be easily miniaturized. Their high aspect ratio due to the comb-like electrode structure increases their sensing surface area and leads to higher sensitivity and high signal-to-noise ratio (SNR) [7,11]. Due to its benefits of low power consumption, high sensitivity, low limit of detection, wide linear response range, simple miniaturization, and the possibility to function without a reference electrode, EIS can enable many new applications, where portability is an important requirement [5,8,15]. Therefore, there is an increasing interest in its application in sensing. This can be seen in Figure 1, which shows that the number of publications in Google Scholar on “EIS” combined with “sensor” has increased rapidly over the past years.

In most publications, the impedimetric sensor response over frequency is complex and not well understood. Apart from standard circuits such as the Randles circuit for faradaic sensors [6,14], equivalent circuits are neglected or not interpreted [12,13], or the derived equivalent circuits differ a lot for similar sensors, as shown in [16]. Some articles start investigating and validating the equivalent circuit of their sensor in more detail, such as [16,17]. Some articles help to choose a relevant equivalent electrical circuit by providing a guideline and comparing different approaches such as [18,19]. However, especially for non-faradaic 2-electrode sensors covered by a sensing layer, there is little literature that includes equivalent circuit fitting and the analysis of the fitting values, or there are discrepancies in the fitting results and the theoretical values [20]. Non-faradaic EIS does not involve redox reactions and is a non-destructive method compared to faradaic EIS. Thus, repeated measurements in the same sample are possible, and the sensor degradation over time is decreased [15]. Opposite to faradaic EIS, this method works without diffusion processes. Hence, no Warburg impedance and no charge transfer resistance occur. Instead, the processes at the electrode interfaces are being monitored [10,21,22]. In this article, we focus on non-faradaic and label-free impedimetric sensors. Additionally, we are only considering two-electrode transducer models where no reference electrode is required due to their advantages of simple miniaturization, lower costs, and less drift [5,7,15]. Despite all the positive characteristics of EIS, it is a time-consuming method, as measurements over a wide frequency range are performed. Hence, it is in the interest of every researcher to identify the most relevant frequency ranges, e.g., in terms of sensor sensitivity and selectivity. This can be achieved by interpreting the data using equivalent circuit models and thus understanding the physical and electrochemical mechanisms in each frequency range. By this, sensor application can be improved regarding time and power consumption by only measuring at the most relevant frequencies. However, due to overlapping effects, measurements at one frequency often contain information from other components that are not desired to be measured, even if the frequency is well chosen. This work aims to separate these overlaying effects to make sensor readings more accurate. Furthermore, sensor design can be optimized by correlating sensor dimensions and design parameters to the sensor response.

In this article, we first introduce the manufacturing methods of four sensor topologies and the tools used for sensor measurement and simulation. Then, we analyze the transducer model using analytical calculations in Section 3.1 to show how material choices and sensor geometries influence the sensor capacitance. Here, we take a closer look at the electrodes, the sensing layer, and the substrate, and we explain which properties have the biggest influence on sensitivity. Second, we derive equivalent circuit models for all four sensors and fit them to measurement data. We start with a basic conductivity sensor to derive more complex models from this known equivalent circuit. Lastly, in Section 3.3, we use FEM simulations to validate these equivalent circuit models and to gain a better understanding of the impedimetric sensor response. This article thereby connects impedimetric ion-sensor responses with their corresponding equivalent circuits and validates these circuits with the help of finite element analysis and analytical calculations. To our knowledge, no other work has connected all these validation methods for equivalent circuit interpretation. By these means, this article helps with equivalent circuit design, sensor data interpretation, and geometry optimization for the best sensitivity and selectivity.

## 2. Materials and Methods

### 2.1. Sensor Manufacturing and Characterization

**Figure 2:**
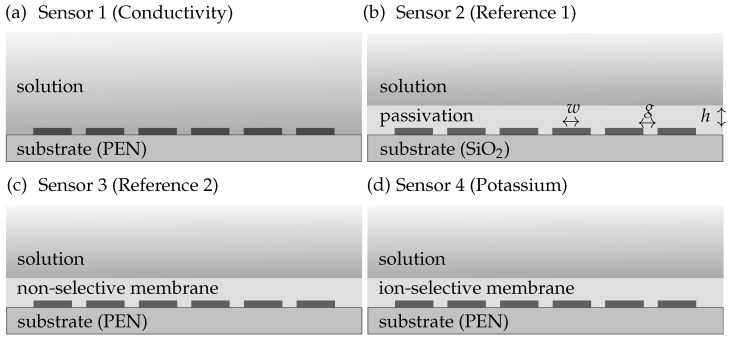
The four sensors topologies including (**a**) a conductivity sensor with Au IDEs on PEN substrate (sensor 1), (**b**) a conductivity sensor with a passivation layer on a wafer substrate (sensor 2), (**c**) a reference sensor with a non-selective membrane (sensor 3), and (**d**) an ion sensor with an ion-selective membrane on PEN (sensor 4).

We manufactured four different sensor topologies, which are depicted in Figure 2. The first topology is a conductivity sensor (see Figure 2a) and consists of 500 μm width and distance gold (Au) IDEs on 100μm polyethylene naphthalate (PEN) foils (Teonex Q51, Q81, and Q83, Pütz Folien). The manufacturing process of the Au electrodes on PEN foil is described in detail in [9]. Sensors 3 and 4 depicted in Figure 2c,d additionally contain a butylacrylate-based ion-selective membrane (ISM) on top of the IDEs (recipe provided in [9]). Sensor 3 is a reference sensor without ionophores, whereas sensor 4 contains 4% of potassium ionophore III. The ISM was dropcasted on the electrodes and cured via UV light.

The electrodes of sensor 2 (see Figure 2b) were made using the top metal layer of a 180nm CMOS process. Thus, the substrate material is silicon (Si) and the electrode material is 880nm thick aluminum–copper alloy (AlCu). The IDEs have a width of 30μm and a distance of 20μm. Additionally, there is a 6μm passivation layer on top of the electrodes, which consists of 2μm silica (${\mathrm{SiO}}_{2}$) with silicon nitride (${\mathrm{Si}}_{3}N_{4}$) and 4μm polyamide with a total relative permittivity of $\varepsilon_{r}\approx 4$.

For EIS measurements, a glass ring was glued on top of the ISMs to provide a cavity for a standardized electrolyte height of 10mm and a volume of 250μL. The impedimetric sensors were characterized using a Gamry Interface 1010E potentiostat, which is optimized for high impedance ranges. EIS measurements were carried out at frequencies of 1MHz–100MHz with AC voltages of 10 and 250mV.

### 2.2. Sensor Models and Simulations

We gradually developed equivalent circuit models of the sensors based on their impedance responses, starting with the most simple sensor (IDEs in air) and adding to them layer by layer (i.e., electrolyte and ISM). Circuit fitting to the measured data was performed with Gamry Echem Analyst software, version 7.9.0 (Simplex Model).

For analytical calculations of the sensors’ capacitances, we employed the parallel partial capacitance (PPC) method for sensor models without a membrane and with a membrane in air, as well as the serial partial capacitance (SPC) method for sensors with a membrane immersed in electrolytes. Both methods are described in more detail in [23,24]. We simplified the equations and used a MATLAB^®^ R2023b script for parameter sweeps.

We used COMSOL Multiphysics^®^ software, version 6.2 (electrostatic and AC/DC module) for finite element (FEM) simulations of the sensors. The simulation parameters are shown in Table 1 as the tested ranges for parameter sweeps and the fixed values.

We then compared the fitted values for the passive components of the equivalent circuit (i.e., R, C) to the theoretical values obtained from FEM simulations and analytical calculations to verify coherent results.

## 3. Results

### 3.1. Transducer Model—Materials, Geometry, and Capacitance

The transducer layout of an impedimetric sensor is crucial for the achievable sensitivity of the sensor. It is a trade-off between high sensitivity and selectivity and low costs and simple sensor prototyping. Therefore, in this section, we investigate how different substrate materials and electrode geometries influence the sensitivity. Sensor capacitances are calculated analytically with SPC and PPC methods and simulated in COMSOL Multiphysics^®^ (electrostatic model) dependent on sensor variables such as electrode dimensions or substrate and membrane materials and thicknesses. With this, we calculate and compare the theoretic static capacitances of our four sensor topologies (Figure 2) in different environments and study how they can be adjusted.

#### 3.1.1. Interdigitated Electrodes

**Figure 3:**
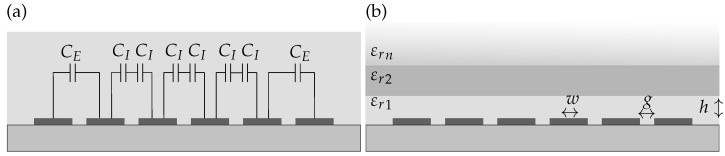
Cross-section of an interdigitated electrode array with 6 fingers with (**a**) the external and internal capacitances $C_{E}$ and $C_{I}$ and (**b**) the declaration of $\varepsilon_{r}$ for the different layers on top of the electrodes.

For interdigitated electrodes in air, the sensor capacitance *C* can be analytically derived by

(2) $$C=\left(N-3\right)\frac{C_{I}}{2}+2\frac{C_{I}C_{E}}{C_{I}+C_{E}},$$

where *N* is the number of interdigitated fingers, $C_{I}$ is the capacitance of the inner electrode pairs and $C_{E}$ the capacitance of the two external electrode pairs, as depicted in Figure 3a [23,24].

$C_{I}$ and $C_{E}$ are functions of the metallization ratio η and the the height-to-width ratio *r* defined by

(3) $$\eta =\frac{w}{w+g}$$

and

(4) $$r=\frac{2h}{w+g},$$

where *w* and *g* are electrode width and gap and *h* is the corresponding layer height. Hence, *r* is a variable that is dependent on the respective layer. For increasing relative permittivities of the layers with distance to the electrodes (i.e., ${\varepsilon_{r}}_{1}<{\varepsilon_{r}}_{2}$) the serial partial capacitance (SPC) method

(5) $$\frac{1}{C_{cell}}=\frac{1}{L}\sum_{i=0}^{n-1}\left(\frac{1}{\varepsilon_{i}}-\frac{1}{\varepsilon_{i+1}}\right)\cdot \frac{1}{\kappa_{cell}^{c}\left(\eta ,r_{i}\right)}+\frac{1}{\varepsilon_{n}}\cdot \frac{1}{\kappa_{cell}^{c}\left(\eta ,r_{n}\right)}$$

must be used, and for ${\varepsilon_{r}}_{1}>{\varepsilon_{r}}_{2}$ the parallel partial capacitance (PPC) method

(6) $$C_{cell}=L\sum_{i=0}^{n-1}\kappa_{cell}^{c}\left(\eta ,r_{i}\right)+\varepsilon_{n}\kappa_{cell}^{c}\left(\eta ,r_{n}\right)$$

applies. For sensor 1, i.e., sensors without a membrane on top of the IDEs (see Figure 2a), assumption I [24] implies that the layer on top of the electrodes (e.g., air or solution) has an infinite height $r_{air/sol}\to \infty$ leading to

(7) $$\kappa_{cell}^{C}\left(\eta ,r_{sol/air}\right)=\frac{K(k^{\prime })}{K(k)},$$

where

(8) $$k^{C_{I}}=\sqrt{sinh^{2}\left(j\frac{\pi \eta }{2}\right)+1},$$

(9) $$k^{C_{E}}=\sqrt{-\frac{4\eta }{{\left(\eta +1\right)}^{2}}+1},$$

and

(10) $$k^{\prime }=\sqrt{1-k^{2}}.$$

In this case, $\kappa_{cell}^{C}$ is independent of the height-to-width ratio *r*. Solving Equation (3) for our IDES design of w=g=500μm, we obtain η=0.5. Inserting these results in Equations (8)–(10), and solving the circular integrals $K(k^{\prime })$ and K(k), we obtain the fixed values $\kappa_{cell}^{C_{E}}\left(\eta ,r_{sol}\right)=1.640068238863158$ and $\kappa_{cell}^{C_{I}}\left(\eta ,r_{sol}\right)=1$.

**Figure 4:**
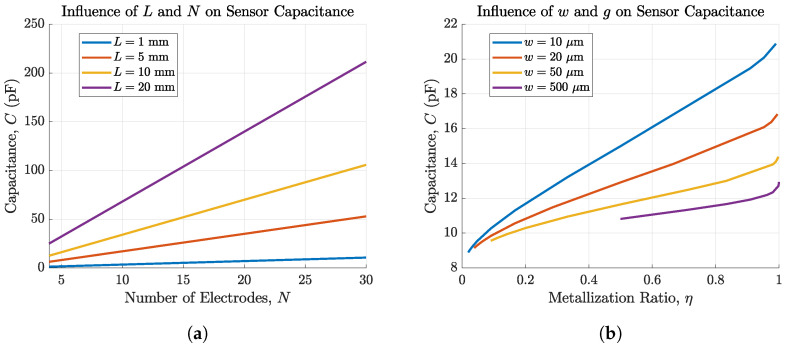
Capacitance of IDEs depending on (**a**) the number of electrode fingers *N* and the length of these fingers *L* and (**b**) the metallization ratio η for *w* ranging from 10–500μm and *g* ranging from 0.1–500μm. The capacitance can be increased by more than 230% for w=10μm.

Figure 4 shows the dependency of the sensor capacitance *C* on the electrode parameters *L*, *N*, *w*, and *g* analytically calculated with our MATLAB^®^ scripts based on Equations (2)–(10). Figure 4a shows that *C* increases with an increasing number and length of fingers. The slope of increase with the number of fingers is steeper for higher values of *L*. This reveals that the finger length has a bigger impact on the sensor capacitance than the number of fingers. For these calculations, *w* and *g* were set to 500μm. Additionally, Figure 4b shows consistency with [7] that the sensors’ capacitance can be increased with a higher metallization factor η, hence the bigger the electrode width and the smaller the gap, the higher the sensitivity. For w=10μm, the capacitance can be increased for more than 230% from 8.89pF for η=0.02 to 20.88pF for η=0.99. Electrode fingers were fixed to a length of 5.5mm and a number of 6 corresponding to our sensor dimensions. As the electrode height is very small (50–100nm) compared to their width 10–500μm, its influence on the capacitance can be neglected [23].

#### 3.1.2. Passivation and Sensing Layer

Sensors 2–4 contain an additional insulating layer on top of the electrodes. For sensor 2, this is a passivation layer with ${\varepsilon_{r}}_{mem}\approx 4$ and for sensors 3 and 4 it is the reference and ion-selective membrane with an at-this-point-unknown relative permittivity ${\varepsilon_{r}}_{mem}$, estimated to be between 4–20. For measurements in air (${\varepsilon_{r}}_{air}=1$), their capacitance must be calculated using the PPC method (see Equation (6)) since ${\varepsilon_{r}}_{mem}>{\varepsilon_{r}}_{air}$. For sensors 2–4 in solution (${\varepsilon_{r}}_{sol}=80$), the SPC method (see Equation (5)) must be used since ${\varepsilon_{r}}_{mem}<{\varepsilon_{r}}_{sol}$. The SPC method leads to

(11) $$\frac{C_{E}}{\varepsilon_{0}}=L\left(\frac{{\varepsilon_{r}}_{sol}{\varepsilon_{r}}_{mem}\kappa_{cell}^{C_{E}}\left(\eta ,r_{mem}\right)\kappa_{cell}^{C_{E}}\left(\eta ,r_{sol}\right)}{\kappa_{cell}^{C_{E}}\left(\eta ,r_{mem}\right){\varepsilon_{r}}_{mem}+\kappa_{cell}^{C_{E}}\left(\eta ,r_{sol}\right)\left({\varepsilon_{r}}_{sol}-{\varepsilon_{r}}_{mem}\right)}+\kappa_{cell}^{C_{E}}\left(\eta ,r_{sub}\right){\varepsilon_{r}}_{sub}\right)$$

for $C_{E}$ and

(12) $$\frac{C_{I}}{\varepsilon_{0}}=L\left(\frac{{\varepsilon_{r}}_{sol}{\varepsilon_{r}}_{mem}\kappa_{cell}^{C_{I}}\left(\eta ,r_{mem}\right)\kappa_{cell}^{C_{I}}\left(\eta ,r_{sol}\right)}{\kappa_{cell}^{C_{I}}\left(\eta ,r_{mem}{\varepsilon_{r}}_{mem}\right)+\kappa_{cell}^{C_{I}}\left(\eta ,r_{sol}\right)\left({\varepsilon_{r}}_{sol}-{\varepsilon_{r}}_{mem}\right)}+\kappa_{cell}^{C_{I}}\left(\eta ,r_{sub}\right){\varepsilon_{r}}_{sub}\right)$$

for $C_{I}$. It can be seen that the capacitance is a function of the relative permittivities $\varepsilon_{r}$ of the membrane, the substrate, and the electrolyte, as well as the metallization ratio η and the height-to-width-ratio *r*. Using assumption II for finite height layers and SPC method (Dirichlet b.c. [24]), where the height-to-width ratio of the membrane $r_{mem}\to 0$ and the substrate $r_{sub}\to 0$ and for electrodes with w=g and therefore η=0.5, $\kappa_{cell}^{C_{E}}$ and $\kappa_{cell}^{C_{I}}$ can be simplified to

(13) $$\kappa_{cell}^{C_{E}}\left(\eta ,r_{mem/sub}\right)=\frac{2\eta }{r_{mem/sub}}+1=1+\frac{1}{r_{mem/sub}}$$

and

(14) $$\kappa_{cell}^{C_{I}}\left(\eta ,r_{mem/sub}\right)=\frac{\eta }{r_{mem/sub}}+0.5=\frac{\kappa_{cell}^{C_{E}}\left(\eta ,r_{mem/sub}\right)}{2}$$

respectively. The same assumptions (w=g, η=0.5, $r_{sub}\to 0$, $r_{mem}\to 0$) for finite height layer using the PPC method (Neumann b.c. [24]) lead to the simplifications

(15) $$\kappa_{cell}^{C_{E}}\left(\eta ,r_{mem/sub}\right)=\frac{r_{mem/sub}}{1-\eta }=2\;r_{mem/sub}$$

and

(16) $$\kappa_{cell}^{C_{I}}\left(\eta ,r_{mem/sub}\right)=\frac{1}{2}\left(\frac{r_{mem/sub}}{1-\eta +r_{mem/sub}}+\frac{r_{mem/sub}}{1-\eta }\right)=\frac{r_{mem/sub}}{1+2\;r_{mem/sub}}+r_{mem/sub}.$$

**Figure 5:**
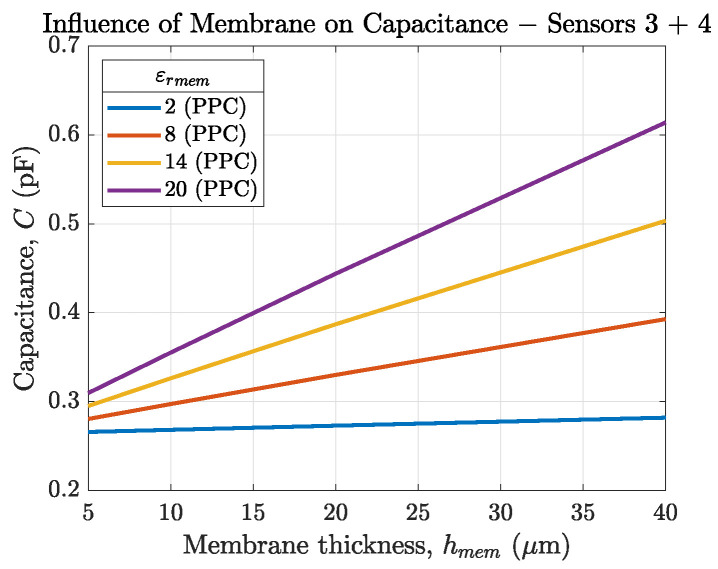
Capacitance of sensor 3 and 4 depending on the membrane thickness for ${\varepsilon_{r}}_{mem}$ ranging from 2 to 20. The sensor is in air, therefore the PPC method is used (${\varepsilon_{r}}_{mem}>{\varepsilon_{r}}_{air}$).

**Figure 6:**
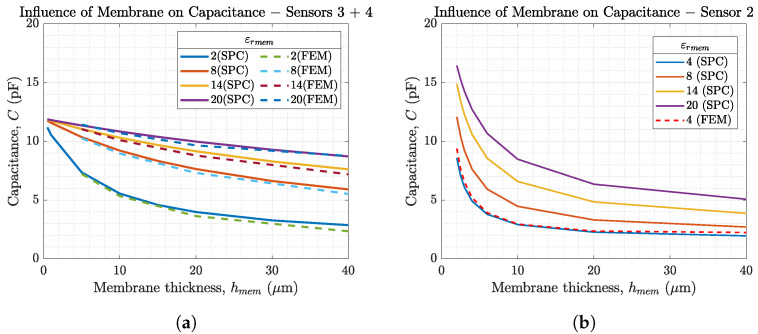
Capacitances of our sensor topologies 2–4 depending on the membrane thickness for ${\varepsilon_{r}}_{mem}$ ranging from 2 to 20. The SPC method is used as there is an electrolyte on top of the membrane (${\varepsilon_{r}}_{mem}<{\varepsilon_{r}}_{sol}$). In (**a**) analytical results (–) are compared to FEM simulations (- -) for sensor 3 and 4. (**b**) shows the influence of membrane parameters for sensor 2.

Figure 5 and Figure 6a compare the sensor capacitances of our sensors 3 and 4 in an environment of air (${\varepsilon_{r}}_{air}=1$, PPC) and solution (${\varepsilon_{r}}_{sol}=80$, SPC). Figure 5 shows that for our sensor layout (N=6,L=5.5mm) the changes in sensor capacitance depending on ${\varepsilon_{r}}_{mem}$ is below 1pF and therefore not measurable. For this reason, and due to the sensors’ aimed application in electrolytes, the SPC method shown in Figure 6 is preferred.

Figure 6 helps to understand the influence of membrane permittivity and membrane thickness on the sensor capacitance, which is increased by thinner membranes and higher ${\varepsilon_{r}}_{mem}$. Due to the serial combination of the membrane capacitance $C_{mem}$ and the solution capacitance $C_{sol}$ the overall sensor capacitance is calculated using

(17) $$C=\frac{C_{mem}C_{sol}}{C_{mem}+C_{sol}}$$

and thus the smaller capacitance value is dominating. The general equation for capacitance $C=\varepsilon \frac{A}{d}$ shows that $C_{mem}<<C_{sol}$. This implies that the membrane parameters have a much larger effect on the sensor capacitance than the solution. Additionally, the changes in ${\varepsilon_{r}}_{sol}$ are comparably small <|1| for ion concentration changes below 100mmol/L [25]. Therefore, changes in ${\varepsilon_{r}}_{sol}$ due to changes in ion concentration are neglected in this article. This is a wanted effect, since we want to extract information about the analyte from the capacitance change of the membrane. From this follows that the ISM is the dominant layer that determines sensor performance. Figure 6a additionally compares the analytically calculated sensor capacitance vs. the sensor capacitances obtained from FEM simulations. The figure shows the good agreement of both methods. Due to its faster computation time, the analytical calculations in MATLAB^®^ are used for further investigations. Figure 6b shows the dependency on the membrane parameters for sensor 2 on a silicon wafer with w=30μm, g=20μm, L=1.5mm, N=30, ${\varepsilon_{r}}_{sub}=3.9\;$ (SiO_2_), and $h_{sub}=300\;\mu$m. By comparing it to Figure 6a, it can be seen that electrode dimensions have a significant influence on the membrane parameters. Therefore, the membrane thickness should always match the IDES layout. This effect is further investigated later in this article.

#### 3.1.3. Substrate

**Figure 7:**
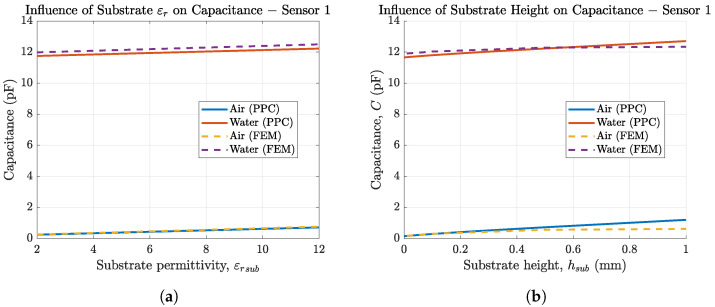
(**a**) IDES capacitance (sensor 1) in air and solution as a function of the substrate permittivity simulated in COMSOL Multiphysics (- -) and calculated in MATLAB (–) and (**b**) capacitance of sensor 1 in air depending on substrate thickness.

As we are using two different substrate materials for our sensors, we investigated the influence of the substrate material on the sensor capacitance. The results are depicted in Figure 7a for sensor 1 employing FEM simulations as well as PPC calculations. Both methods match very well. A higher substrate permittivity leads to higher sensor capacitance, but the change in capacitance is small since the relative permittivity of the substrate must be low for it to be insulating or at least be a dielectric material. As dielectric media get polarized with AC signals, they lead to losses and should therefore be avoided. Furthermore, the influence of ${\varepsilon_{r}}_{sub}$ is small compared to ${\varepsilon_{r}}_{sol}$ due to the parallel connection for both capacitances ($C=C_{sol}+C_{sub}$) and the fact that $C_{sol}>>C_{sub}$, since ${\varepsilon_{r}}_{sol}>>{\varepsilon_{r}}_{sub}$ and $h_{sol}>>h_{sub}$.

Figure 7b shows that thicker substrates increase the overall sensor capacitance. The small differences in FEM and PPC for higher substrate thickness stem from assumption II (finite length approximation of $h_{sub}$) in PPC, which is less accurate for high substrate thickness. In addition to the fact that their influence on the sensor capacitance is neglectable, substrate permittivity and substrate thickness often cannot be adjusted much, since they are material properties or depend on the use case, e.g., for the sensor to be flexible.

### 3.2. Measurement Results and Equivalent Circuit Models

**Figure 8:**
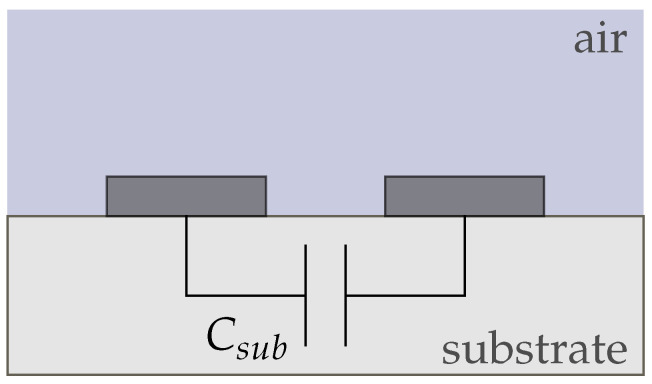
Equivalent circuit of conductivity sensor (sensor 1) in air. The behaviour is purely capacitive and dominated by $C_{sub}$.

We derived four equivalent circuit models for our conductivity sensor in air and electrolyte (sensor 1), the wafer electrodes with passivation (sensor 2), and the membrane-covered sensors 3 and 4. Figure 8 shows the equivalent circuit for sensor 1 in air, which only consists of the substrate capacitance $C_{sub}$ and the capacitance of air $C_{air}$. Since both capacitances are in parallel, the smaller capacitance $C_{air}$ can be neglected and the circuit can be simplified to $C_{sub}$. Due to the very low conductivities of air and the substrate, we did not include any resistances between the electrodes.

**Figure 9:**
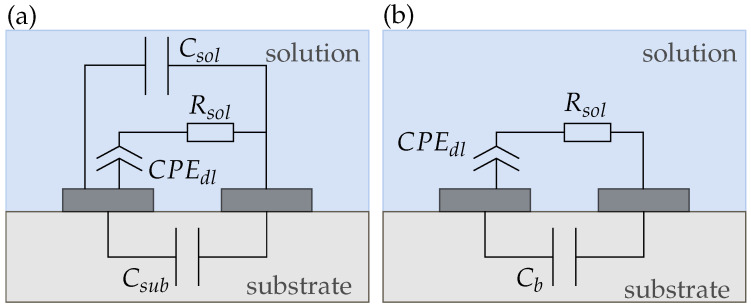
(**a**) Complete and (**b**) simplified equivalent circuit of a conductivity sensor (sensor 1) in solution. The capacitances of each layer can be added up to a background capacitance $C_{b}$.

When we add a solution (e.g., DI water containing K^+^ ions) on top of the electrodes the circuit complexity increases as shown in Figure 9a. The electrolyte adds a capacitance $C_{sol}$ and due to the charge mobility in the solution a resistance $R_{sol}$ to the circuit. Additionally, a double-layer capacitance $C_{dl}$ builds up at the electrode|solution interface [16]. As both electrodes are identical their $C_{dl}$ can be summarized. Instead of an ideal $C_{dl}$, commonly a constant phase element (CPE) is used to model the double layer effect as it represents real interfaces with surface heterogeneity [26]. The impedance of a CPE is calculated using

(18) $$Z_{CPE}=\frac{1}{Y_{0}{\left(j\omega \right)}^{\alpha }}$$

where 0<α<1. For α=1, it acts like an ideal capacitor and $Y_{0}=C$, while for α=0 it becomes a resistor with $Y_{0}=R$ [8]. For circuit simplifications, the parallel capacitances $C_{sol}$ and $C_{sub}$ can be added up to a background capacitance $C_{b}$ as shown in Figure 9b. $C_{b}$ is dominated by the larger solution capacitance $C_{sol}$ due to their parallel connection. This equivalent circuit aligns with other literature reported for electrodes in direct contact with the electrolyte [5,16]. However, in most publications, $C_{b}$ is neglected and only $C_{dl}$ and $R_{sol}$ are used for equivalent circuit modeling [14,15].

**Figure 10:**
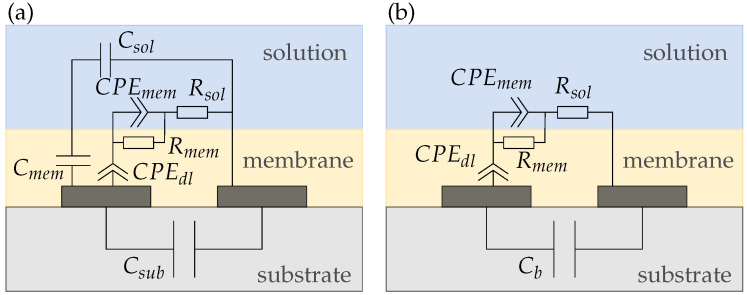
(**a**) Complete and (**b**) simplified equivalent circuits of a reference (sensor 3) and potassium sensor (sensor 4), respectively.

The equivalent circuit for sensors with a membrane, coating both electrodes (i.e., sensors 3 and 4) is shown in Figure 10. The membrane adds a capacitance $C_{mem}$ and a resistance $R_{mem}$ to the circuit. Moreover, a second $CPE_{dl}$ develops at the membrane|solution interface. Again, Figure 10b shows the simplified equivalent circuit with the capacitances summarized to a background capacitance $C_{b}$.

**Figure 11:**
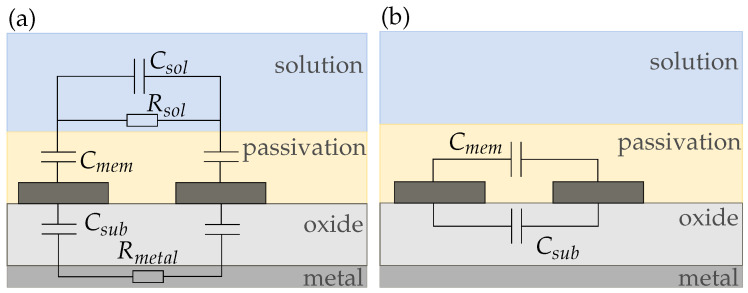
(**a**) Complete and (**b**) simplified equivalent circuits of a passivated sensor on silicon (sensor 2). If the passivation is too thick compared to the electrode dimensions, all of the electric field is inside the membrane and the sensor response gets purely capacitive.

Figure 11 shows the equivalent circuit for sensor 2. In Figure 11a, we propose an equivalent circuit for electrodes with a very thin passivation layer below 2μm. For our sensor, however, the passivation thickness is 6μm. From Figure 6, it can be seen that in this case, the sensor capacitance does not change much with increasing membrane thickness. Therefore, we assume that the sensor capacitance is dominated by the membrane capacitance and we can simplify the equivalent circuit to Figure 11b such that the sensor is purely capacitive and due to the hydrophobic nature of the passivation independent of the solution.

**Figure 12:**
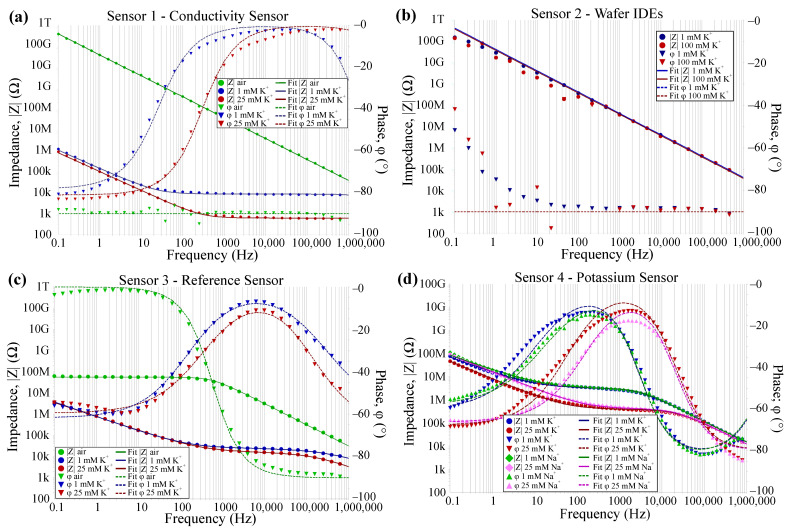
Sensor measurements in air and 1 and 25 mmol/L K^+^ (·) and equivalent circuit fitting results (–) for (**a**) conductivity, (**b**) passivated IDEs on SiO_2_, (**c**) reference sensor, and (**d**) potassium sensor [27].

The measurement results for 1 and 25 mmol/L K^+^ in DI water for all four sensor topologies are depicted in Figure 12. More extensive measurements of sensor 4, including a calibration curve, are presented in [9]. The dotted lines in Figure 12 are the measurement results and the solid lines represent the respective circuit fitting results employing the afore-introduced equivalent circuits. Both show very good agreement in magnitude |Z| and phase φ for all sensors. This supports our proposed equivalent models. Furthermore, the equivalent circuit fitting delivers values for the electric components used in the equivalent circuit. This helps to identify and understand the influence of ionic strength on the individual sensor responses.

The values of the circuit fitting are shown in Table 2, which summarizes the effect of the ionic strength on the circuit components for all four sensor topologies. For the conductivity sensor, the solution resistance decreases with increasing ion concentration due to the increased charge mobility of the ions. Additionally, the double-layer capacitance increases because the double-layer thickness decreases with increasing ion concentration [28]. Both effects are not selective. The higher conductivity of potassium compared to sodium is due to their smaller hydrate shell [29].

For the reference sensor, $CPE_{dl}$ is independent of ion concentrations due to the lack of ionophores in the membrane. However, the membrane resistance is decreased from 24–26kΩ to 12–15kΩ respectively for changing ionic strength. We neglected $R_{sol}$, as the response of this component is only visible in higher frequency ranges and therefore led to fitting errors. When measuring the dry, unconditioned sensor in air, we measured a membrane resistance of 25–55MΩ, which can be attributed to the conductive salt in the membrane composition (compare to [9]) and a $C_{b}$ of 5pF. Even though $R_{M}$ and $CPE_{M}$ show a slight sensitivity, none of the sensor components are selective.

As it can be seen from the measurements of the potassium sensor in air, the presence of the ionophore increases the resistance of the dry membrane compared to the reference sensor such that it cannot be measured in the tested frequency range. From this follows that in air, we only measure a sensor capacitance of 4pF. This is the resolution limit of our laboratory potentiostat. The calculated $C_{b}$ is <0.5pF (see Figure 6a in Section 3.1.2).

Due to the overlaying effects of the capacitances and the membrane|solution double-layer, they cannot be clearly distinguished from one another. Therefore, we neglected $C_{b}$ in our circuit fitting. From this follows that $CPE_{M}$ cannot be attributed to one effect but is assumed to be a combination of multiple capacitive components. From Table 2 we see that $R_{M}$, $CPE_{M}$, and $CPE_{dl}$ all depend on the ionic strength of the electrolyte. However, only the change in $CPE_{dl}$ is 11nF from 1mmol/L to 25mmol/L K^+^, compared to no change for the same Na^+^ concentrations. Comparing these results to $CPE_{dl}$ of sensor 3, where no dependency on ionic strength is visible, it suggests that the double-layer formation at the electrode surface relies on the presence of ionophores. Therefore, $CPE_{dl}$ shows the highest selectivity for K^+^ ions and measurements at low frequencies (e.g., 100mHz) should be chosen for selectivity measurements despite their long duration. The drift of the potassium sensors in this frequency domain is low [9], and as shown in [27]; especially, the phase response is very fast and repeatable due to the capacitive nature of the sensor in this frequency domain.

For the electrodes on silicon, we see that the capacitance is independent of ionic strength, suggesting that the passivation layer is too thick and no ions can penetrate the membrane. This aligns with Figure 6b. To further validate this theory we measured at higher ion concentrations of 100mmol/L instead of 25mmol/L. For all concentrations, the measured $C_{b}$ is 4.1pF, which aligns with our calculations depicted in Figure 6b.

### 3.3. Measurement Results and FEM Simulations

In this section, we present the FEM simulation results of our sensors to further validate the developed equivalent circuits and give recommendations for sensor design optimizations. For this reason, the required membrane thickness in dependency of the electrode distances is examined. With the help of FEM simulations, sensor parameters such as dimensions and material properties (e.g., permittivity and conductivity) can be swept iteratively to evaluate the influence of single parameters on the sensor response. Therefore, we are not restricted to assumptions like in Section 3.2, where overlaying effects cannot be differentiated.

#### 3.3.1. Validation of Equivalent Circuit Models

**Figure 13:**
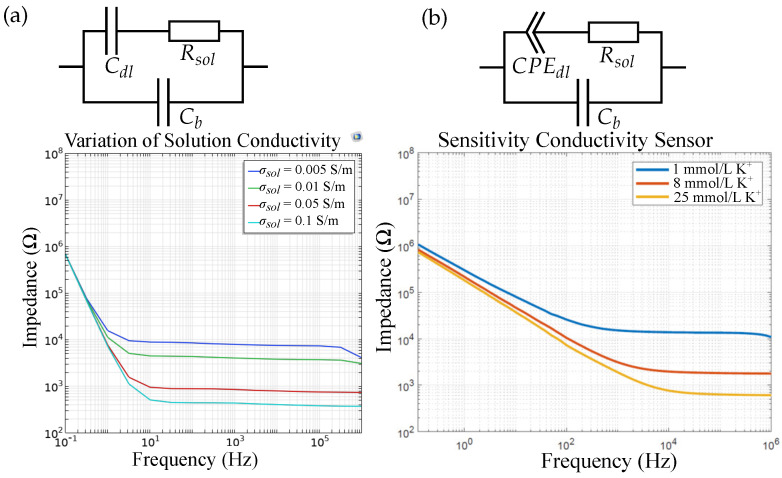
(**a**) FEM simulation results and the respective equivalent circuit vs. (**b**) measurement results for a conductivity sensor (sensor 1). The double-layer capacitance is replaced by a CPE for real sensors. The change in ion concentration can be simulated by a change in solution conductivity.

For sensor 1, Figure 13 shows the (a) simulated and (b) the measured impedimetric sensor response for varied ion concentration in DI water, as well as the corresponding equivalent circuits. We measured the conductivity sensor in 1,8, and 25mmol/L K^+^. We can see that the ion concentration can be simulated as a variation in solution conductivity, or solution resistance $R_{sol}$, respectively. The solution conductivity is a function of the ion concentration and is dependent on the molar conductivity of the specific ion. Typical values are about 10mS m^2^ mol^−1^ and vary with the ion and its concentration [29]. The theoretical solution conductivity of 1mmol/L K^+^ is, for example, 0.015S/m. The double layer is simulated as a thin (1–10nm [28]) insulating layer on top of the IDEs. The difference in the slope of the impedance in that the low-frequency region can be explained due to the imperfect electrode surface of the real sensor compared to the simulation model. Due to the surface roughness and impurities, we obtain a $CPE_{dl}$ for the measured sensor, compared to a real capacitance due to the $C_{dl}$ in FEM simulations. As the changes in solution permittivity are relatively small (≈−1) for these ion concentration ranges [25], the changes in ${\varepsilon_{r}}_{sol}$ are neglected in simulations and the sensor capacitance is not influenced by varying ion concentrations.

**Figure 14:**
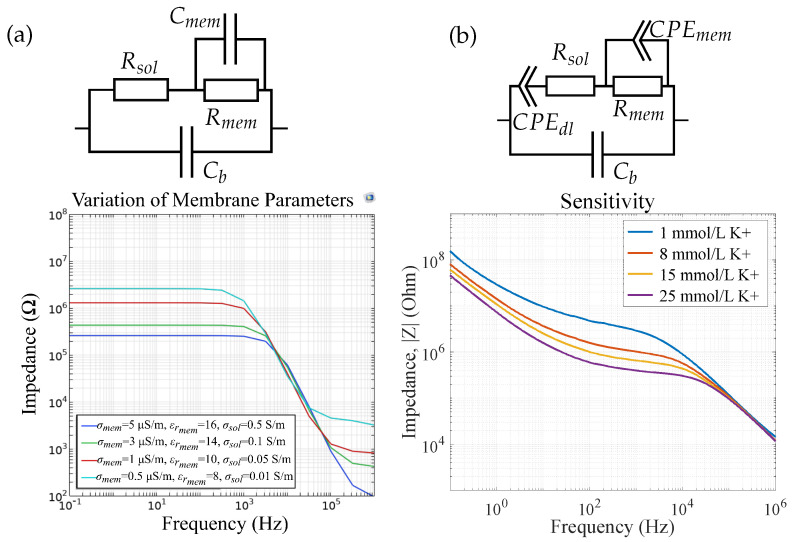
(**a**) FEM simulation results and the respective equivalent circuit vs. (**b**) measurement results for a potassium sensor (sensor 4), reprinted with permission from [9]. The double-layer capacitance is neglected in simulations. Changes in ion concentrations can be modeled by changes in solution conductivity and membrane conductivity and permittivity.

For sensor 4, i.e., a sensor with an ISM and an electrolyte on top of the IDES, the FEM simulation results are depicted in Figure 14a. To improve the simulation time, we first neglected the double-layer capacitance formed at the electrode|membrane interface and varied only the membrane conductivity $\sigma_{mem}$, the membrane permittivity ${\varepsilon_{r}}_{mem}$, and the solution conductivity $\sigma_{sol}$ as these material properties are expected to be affected by varying ion concentration in the electrolyte. Figure 14b shows the corresponding measurement results of a potassium sensor (sensor 4) which align with the simulation results. The change in $R_{mem}$ and $C_{mem}$ from the equivalent circuit can be referred to as a change in $\sigma_{mem}$ and ${\varepsilon_{r}}_{mem}$ respectively due to increasing ion concentration. This shows that both $\sigma_{mem}$ and ${\varepsilon_{r}}_{mem}$ are dependent on the ion concentration inside the membrane due to the presence of ionophores.

**Figure 15:**
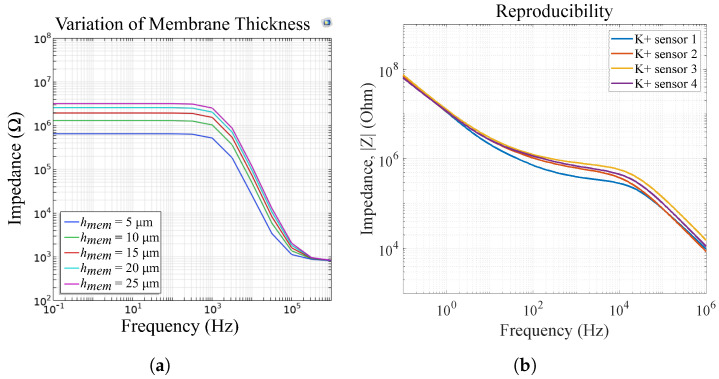
(**a**) FEM simulations of varying membrane thicknesses for sensor 4 and (**b**) measurement results of four potassium sensors in 25mmol/L K^+^, reprinted with permission from [9]. The reproducibility errors can be attributed to differences in membrane thickness due to the manufacturing process via drop casting.

Figure 15 shows the influence of the membrane thickness on the impedance magnitude of sensor 4. We observe a change in $R_{mem}$ and $C_{mem}$, respectively. The change in $C_{mem}$ can be explained with Equations (4) and (8)–(14), where $r_{mem}$ is a function of $h_{mem}$. In Figure 15b, we measured four potassium-selective sensors in 25mmol/L K^+^ and we observe the same variations as in our FEM simulations. Therefore, we can attribute the impedance deviations in differing sensors to differences in their membrane height, which occur due to the ISM deposition via drop-casting. In [9], we show that our ISMs can also be ink-jet printed. This method can enable the improved reproducibility of membrane thicknesses, leading to reproducible sensor results and thus eliminating the need for sensor calibration.

**Figure 16:**
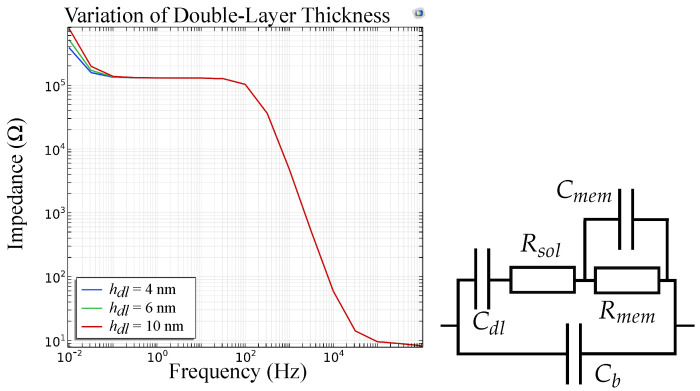
FEM simulations and equivalent circuit of an ion sensor with $C_{dl}$ for varying thickness of the double-layer. The effect is visible at low frequencies (<1 Hz).

The FEM simulations for a sensor model including a double-layer capacitance at the electrode|membrane interface are depicted in Figure 16 for different thicknesses of the double layer. This shows that for increasing thickness of the double layer, in low frequencies the graph is shifted to the left. This aligns with our measurements as the double-layer thickness decreases with increasing ion concentrations. We assume that the ionophores inside the ISM facilitate the travel of the target ion to the electrode|membrane interface and thereby enable selective measurements in this frequency range. Figure 16 now shows the same course of the graph as measurements of our potassium selective sensor (Figure 14b), thus validating the suggested equivalent circuits.

The step-wise FEM simulations of our sensor validate the developed equivalent circuits and help to increase the understanding of the sensor response. In sensor development, this process can support the improvement of sensor sensitivity and selectivity by understanding which physical or chemical process is responsible for the sensor response and what is the optimum measurement frequency to monitor this process. Additionally, it can help with identifying reasons for sensor failure which will be shown in the next section.

#### 3.3.2. Sensor Design Optimizations

**Figure 17:**
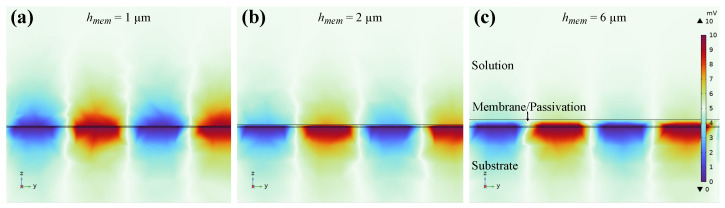
Cross-section of sensor 2 in COMSOL Multiphysics and electric potential plot for (**a**) 1μm, (**b**) 2μm, and (**c**) 6μm passivation layer thicknesses on top of the electrodes. Only for 1μm a significant portion of the electric field reaches into the electrolyte.

After validating the sensor theory we want to examine how the sensor response can be optimized by design alterations. First, the membrane height has a big influence on the impedance response as can be seen, e.g., in Figure 15 and has to match with the electrode dimensions. For IDES where both electrodes are covered by a selective membrane, sensor selectivity can be increased if most of the electric field is inside of the membrane. This results in an increased measurement of membrane parameters, instead of changes in electrolyte conductivity as shown in Section 3.2. On the contrary, thicker membranes lead to slower response times of the sensor. Therefore, we recommend to optimize the thickness depending on the purpose of the membrane. Additionally, a passivation layer that is too thick can lead to a lack of sensitivity and thus to sensor failure. This effect can be seen in Figure 17. Here, the electric potential is shown for passivation layer thicknesses of 1–6μm. It reveals that already for layer thicknesses of 2μm almost no potential reaches into the electrolyte (top layer) and thus for the given passivation thicknesses of approximately 6μm we only measure the sensor capacitance of 4.1pF and no effect of ion concentration changes in the electrolyte.

**Figure 18:**
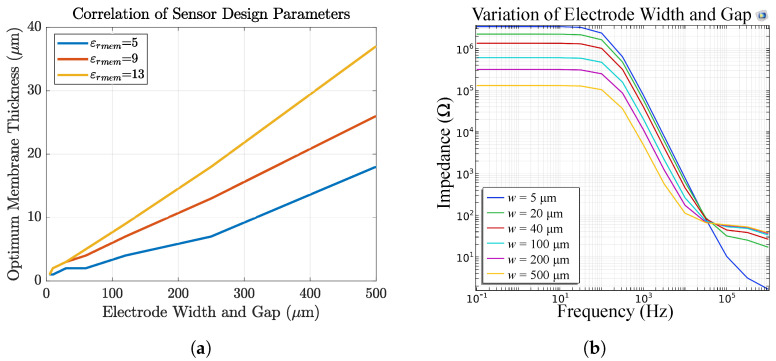
(**a**) The optimum membrane thickness in dependency of IDEs *w* and *g* and membrane permittivity. With increasing electrode dimensions, the membrane is recommended to be thicker. (**b**) Impedance response of sensor 4 in dependency of IDEs *w* and *g*. The membrane resistance increases with decreasing electrode structures.

Figure 18a shows the dependency of the optimum membrane thickness on the electrode width and gap, assuming that w=g. We defined the membrane thickness to be optimal if, in FEM simulations, more than 60% of the electric field is inside the membrane. This value originates from our experience where a severe change in the sensor signal was observed during measurements when more than 60% of the electric field in FEM simulations is inside the membrane. For a selective membrane optimum means the highest selectivity, whereas for a passivation layer, optimum means a maximum that should not be exceeded if sensitivity is required. It can be seen that the optimum thickness is dependent on the permittivity of the membrane and the IDEs’ design parameters. For example, for our sensor 2, a passivation thickness of only >2 μm leads to sensor failure. For our sensors 3 and 4 the membrane thickness should be between 15–40μm depending on ${\varepsilon_{r}}_{mem}$.

Lastly, Figure 18b shows the influence of electrode *w* and *g* on the sensor impedance. We observe a change in the capacitive as well as in the resistive part of the magnitude. For bigger electrode structures the membrane resistance decreases whereas the solution resistance increases.

## 4. Discussion

In this article, we compared theoretical capacitance calculations, equivalent models, and FEM simulations of four different sensor topologies to their measurement results based on an example use case of ion sensors. The article aims to improve the understanding of the impedimetric sensor response and optimize the design for IDEs-based sensors. We first examined the sensor capacitance of IDEs and compared analytical approaches to FEM simulations. For this, we looked at IDEs in air, in solution, and at IDEs covered with a membrane layer. We investigated how sensor design parameters like membrane and substrate materials and electrode dimensions influence the sensor capacitance. Depending on the application, high capacitance values can increase the sensitivity of the sensor and are therefore desirable [7]. The highest capacitance values can be achieved for sensors with small electrode gaps *g* compared to their width *w* and high electrode length *L* and finger number *N*. For IDEs covered by a membrane, its height and permittivity limit the maximum achievable capacitance. For sensor application in fluids, increasing membrane height and decreasing relative permittivity decrease sensor capacitance. For sensor application in air, increasing membrane height and decreasing relative permittivity increase the sensor capacitance. Analytical capacitance results show a good agreement with FEM simulations and support the validity of our model.

**Figure 19:**
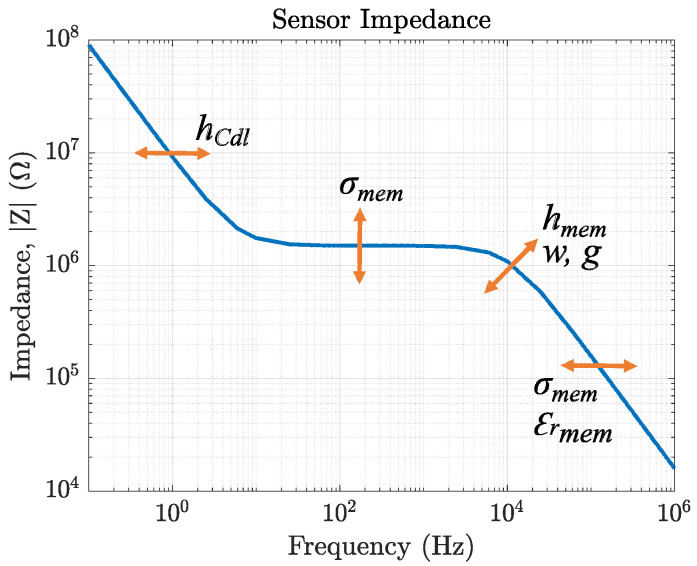
Summary of the most important sensor parameters and their influence on the impedance magnitude for IDEs covered with a membrane. The arrows indicate how the magnitude is shifted if the respective component (i.e., $w,\;g,\;h_{mem},\;h_{Cdl},\;{\varepsilon_{r}}_{mem}$, and $\sigma_{mem}$) is altered.

We then compared the measurement results of our conductivity sensor (sensor 1), passivated sensor (sensor 2), reference sensor (sensor 3), and potassium sensor (sensor 4) to the capacitance calculations by fitting equivalent circuits to their impedance response. The theoretical values match the measurement results. The equivalent circuits of membrane-covered sensors (topologies 3 + 4) in solution are validated by FEM simulations. Here, we can directly see the influence of changing one specific sensor parameter on the impedance response. These results are summarized in Figure 19. This figure reveals the cross-dependencies of all sensor parameters. Moreover, it highlights the need for FEM simulations to be able to distinguish between overlaying effects of design parameters and sensor characteristics. For the example of a sensor covered with a K^+^-selective membrane, the ionic strength of K^+^ in solution influences $\sigma_{mem}$, ${\varepsilon_{r}}_{mem}$, and $h_{Cdl}$ of the sensor (compare Figure 14).

Depending on which sensor component is expected to be the selective element, the sensor design can be adjusted to increase the response of this component. E.g., if the membrane conductivity is expected to be changed by the target analyte, medium and high-frequency response of the sensor are of interest and the IDEs capacitance should be smaller to increase the sensitive frequency range. On the contrary, if the double-layer capacitance is the selective sensor component, the electrode width should be large to increase the area and thereby increase the capacitance and the SNR. Additionally, the resistive part of the impedance magnitude should be low to shift the capacitive part to higher frequencies and save measurement time. If the membrane permittivity is the sensor element of interest, the IDEs capacitance should be large to shift the relevant impedance magnitude to lower-frequency ranges to reduce the effect of parasitic capacitances.

## 5. Conclusions

Within this article, we present EIS measurement results of four sensor topologies for conductivity measurements (sensor 1), reference measurements (sensors 2 and 3), and potassium ion sensing (sensor 4) based on IDEs. We propose a guideline for the design of impedimetric sensors with application in fluids. Equivalent circuit models have been proposed to better extract sensor information from the EIS measurements by improving the understanding of impedimetric sensor responses. Their understanding can enable faster sensor design and increased sensitivity and selectivity of sensor responses. Furthermore, with a better understanding of the impedimetric sensor response, common error patterns like too-thick passivation layers and interface issues (indicated by a capacitive sensor response), too-thin sensing layers (reduced selectivity), or broken membranes (conductivity sensor) can be detected in sito during measurements and defect sensors can be replaced immediately. As recently shown in [30] machine learning algorithms can be used to extract ion type and ion concentration from EIS data of bare electrodes. The combination of our four sensor topologies together can improve the elimination of cross-sensitivities and environmental disturbances (e.g., temperature). Additionally, the differences in selectivity and sensitivity within the frequency range of one sensor can be used for further data extraction. Our simple equivalent circuit models enable better data extraction, which results in improved sensor data.

## Figures and Tables

**Table 1:** Parameter sweep ranges and fixed values used for FEM simulations and analytical calculations.

Parameter	Range	Fixed Value PEN	Fixed Value Si
Substrate conductivity	$10^{-12}–10^{-16}\;$S/m	$10^{-16}\;$S/m	-
Substrate permittivity	1–12	3 ^1^	3.9
Substrate thickness	1–1000μm	100μm	100μm
Solution conductivity	0.01–0.5 S/m	0.05 S/m	-
Solution permittivity	-	80 ^2^	80
Membrane conductivity	$5\times 10^{-6}–10^{-10}\;$S/m	$10^{-7}\;$S/m	-
Membrane permittivity	2–20	9	4
Membrane thickness	1–40μm	10μm	6μm
Electrode width	10–500μm	500μm	30μm
Electrode gap	0.1–500μm	500μm	20μm
Electrode length	1–20mm	5.5mm	1.5mm
Electrode fingers	4–30	6	30
AC voltage	-	250mV	10mV

^1^ Provided value by PEN foil manufacturer, ^2^ based on deionized water at 20 °C.

**Table 2:** Equivalent circuit fitting results.

Component	Air	1 mmol/L K^+^	25 mmol/L K^+^	1 mmol/L Na^+^	25 mmol/L Na^+^
**Conductivity**					
$C_{b}$ (pF)	<4	15	15		
$CPE_{dl}Y_{0}$ (μF)		1.9	3.9		
$CPE_{dl}\alpha$		0.8	0.8		
$R_{sol}$ (kΩ)		4.33	0.21		
**Reference**					
$C_{b}$ (pF)	4.3–5	−	−	−	−
$CPE_{dl}Y_{0}$ (nF)		195	194	197	200
$CPE_{dl}\alpha$		0.79	0.77	0.81	0.81
$R_{M}$ (kΩ)	25–55	24.3	12.1	26	14.8
$CPE_{M}Y_{0}$ (pF)		6.1	31	5.2	22
$CPE_{M}\alpha$		0.64	0.61	0.63	0.64
**Potassium**					
$C_{b}$ (pF)	<4	−	−	−	−
$CPE_{dl}Y_{0}$ (nF)		20	31	16	16
$CPE_{dl}\alpha$		0.67	0.75	0.65	0.74
$R_{M}$ (kΩ)		3000	370	3000	400
$CPE_{M}Y_{0}$ (pF)		34	57	28	54
$CPE_{M}\alpha$		0.94	0.91	0.95	0.91
$R_{sol}$ (kΩ)		4.3 ^1^	0.2 ^1^	4.5 ^1^	0.2 ^1^
**Wafer**					
$C_{b}$ (pF)	4.1	4.1	4.1 ^2^	4.1	4.1 ^3^

^1^ fixed, ^2^ for 100 mmol/L K^+^, ^3^ for 100 mmol/L Na^+^.

## Data Availability

The data presented in this study are available on request from the corresponding author.

## Abbreviations

The following abbreviations are used in this manuscript:

ISM	Ion-selective membrane
FEM	Finite element
CPE	Constant phase element
IDE	Interdigitated electrode
PEN	Polyethylene naphthalate
PPC	Parallel partial capacitance
SPC	Serial partial capacitance

## References

[1] Criscuolo F., Hanitra M., Taurino I., Carrara S., De Micheli G. (2021). All-solid-state ion-selective electrodes: A tutorial for correct practice. IEEE Sens. J..

[2] Golparvar A., Tonello S., Meimandi A., Carrara S. (2023). Inkjet-Printed Soft Intelligent Medical Bracelet for Simultaneous Real-Time Sweat Potassium (K^+^), Sodium (Na^+^), and Skin Temperature Analysis. IEEE Sens. Lett..

[3] Bobacka J., Ivasaka A., Lewenstam A. (1999). Plasticizer-free all-solid-state potassium-selective electrode based on poly(3-octylthiophene) and valinomycin. Anal. Chim. Acta.

[4] Kovács M., Höfler L. (2022). Effect of Kinetic and Thermodynamic Properties of Solid Contact Ion-Selective Electrodes on the Electrochemical Impedance Spectroscopy Response. J. Electrochem. Soc..

[5] Guimerà A., Gabriel G., Prats-Alfonso E., Abramova N., Bratov A., Villa R. (2015). Effect of surface conductivity on the sensitivity of interdigitated impedimetric sensors and their design considerations. Sens. Actuators B Chem..

[6] Zhang W., Spichiger U. (2000). An impedance study of Mg^2+^-selective membranes. Electrochim. Acta.

[7] Gutierrez H.C., Panigrahi S. (2023). Selective Impedimetric Interdigitated Electrode for Sensing Gaseous, Biological, and Inorganic Targets: A State-of-the-Art Review. IEEE Sens. J..

[8] Bertok T., Lorencova L., Chocholova E., Jane E., Vikartovska A., Kasak P., Tkac J. (2018). Electrochemical Impedance Spectroscopy Based Biosensors: Mechanistic Principles, Analytical Examples and Challenges towards Commercialization for Assays of Protein Cancer Biomarkers. ChemElectroChem.

[9] Korek E., Kounoupioti E., Brederlow R. (2023). Manufacturing of Flexible, Impedimetric Potassium Sensors. IEEE Sens. Lett..

[10] Kazemi S.H., Shanehsaz M., Ghaemmaghami M. (2015). Non-Faradaic electrochemical impedance spectroscopy as a reliable and facile method: Determination of the potassium ion concentration using a guanine rich aptasensor. Mater. Sci. Eng. C. Mater. Biol. Appl..

[11] Dudala S., Srikanth S., Dubey S., Javed A., Goel S. (2021). Rapid Inkjet-Printed Miniaturized Interdigitated Electrodes for Electrochemical Sensing of Nitrite and Taste Stimuli. Micromachines.

[12] Day C., Søpstad S., Ma H., Jiang C., Nathan A., Elliott S.R., Hutter T. (2018). Impedance-based sensor for potassium ions. Anal. Chim. Acta.

[13] Aicher M., Grothe H., Wolf B. (2017). A novel thin film impedance Ca ion sensor for drinking water. Sens. Actuators B Chem..

[14] Lazanas A., Prodromidis M. (2023). Electrochemical Impedance Spectroscopy—A Tutorial. ACS Meas. Sci. Au.

[15] Ameer S., Ibrahim H., Yaseen M., Kulsoom F., Cinti S., Sher M. (2023). Electrochemical Impedance Spectroscopy-Based Sensing of Biofilms: A Comprehensive Review. Biosensors.

[16] Tolouei N.E., Ghamari S., Shavezipur M. (2020). Development of circuit models for electrochemical impedance spectroscopy (EIS) responses of interdigitated MEMS biochemical sensors. J. Electroanal. Chem..

[17] Wang Z., Murphy A., O’Riordan A., O’Connell I. (2021). Equivalent Impedance Models for Electrochemical Nanosensor-Based Integrated System Design. Sensors.

[18] Van Haeverbeke M., Stock M., De Baets B. (2022). Equivalent Electrical Circuits and Their Use Across Electrochemical Impedance Spectroscopy Application Domains. IEEE Access.

[19] Harrington D.A., van den Driessche P. (2011). Mechanism and equivalent circuits in electrochemical impedance spectroscopy. Electrochim. Acta.

[20] Freger V., Bason S. (2007). Characterization of ion transport in thin films using electrochemical impedance spectroscopy I. Principles and theory. J. Membr. Sci..

[21] Xu N., Riley D. (2013). Nonlinear analysis of a classical system: The Faradaic process. Electrochim. Acta.

[22] Ibau C., Arshad M., Gopinath S., Nuzaihan M., Fathil M., Shamsuddin S. (2020). Immunosensing prostate-specific antigen: Faradaic vs. non-Faradaic electrochemical impedance spectroscopy analysis on interdigitated microelectrode device. Int. J. Biol. Macromol..

[23] Igreja R., Dias C. (2004). Analytical evaluation of the interdigital electrodes capacitance for a multi-layered structure. Sens. Actuators A Phys..

[24] Blume S., Ben-Mrad R., Sullivan P. (2015). Modelling the capacitance of multi-layer conductor-facing interdigitated electrode structures. Sens. Actuators B Chem..

[25] Hasted J.B., Ritson D., Collie C. (1948). Dielectric Properties of Aqueous Ionic Solutions. Parts I and II. J. Chem. Phys..

[26] Córdoba-Torres P., Mesquita T., Nogueira R. (2015). Relationship between the Origin of Constant-Phase Element Behavior in Electrochemical Impedance Spectroscopy and Electrode Surface Structure. J. Phys. Chem. C.

[27] Korek E., Kounoupioti E., Brederlow R. (2024). Equivalent Circuit Models for Impedimetric Sensors. Proceedings.

[28] Bohinc K., Kralj-Iglič V., Iglič A. (2001). Thickness of electrical double layer. Effect of ion size. Electrochim. Acta.

[29] Atkins P., de Paula J. (2006). Atkins’ Physical Chemistry.

[30] Sagiroglu M.Z., Demirel E.D., Mutlu S. (2024). Accurate ion type and concentration detection using two bare electrodes by machine learning of non-faradaic electrochemical impedance measurements of an automated fluidic system. JEAC.

